# Multisystem inflammatory syndrome in children (MIS-C): Implications for long COVID

**DOI:** 10.1007/s10787-023-01272-3

**Published:** 2023-07-17

**Authors:** Tamás Constantin, Tamás Pék, Zsuzsanna Horváth, Diána Garan, Attila J. Szabó

**Affiliations:** Department of Pediatrics, Semmelweis University, Tűzoltó u. 7-9., Budapest, 1094 Hungary

**Keywords:** COVID, Children, MIS-C, Long-COVID

## Abstract

The COVID-19 pandemic caused by the coronavirus 2 of the severe acute respiratory syndrome (SARS-CoV-2) has significantly affected people around the world, leading to substantial morbidity and mortality. Although the pandemic has affected people of all ages, there is increasing evidence that children are less susceptible to SARS-CoV-2 infection and are more likely to experience milder symptoms than adults. However, children with COVID-19 can still develop serious complications, such as multisystem inflammatory syndrome in children (MIS-C). This narrative review of the literature provides an overview of the epidemiology and immune pathology of SARS-CoV-2 infection and MIS-C in children. The review also examines the genetics of COVID-19 and MIS-C in children, including the genetic factors that can influence the susceptibility and severity of the diseases and their implications for personalized medicine and vaccination strategies. By examining current evidence and insights from the literature, this review aims to contribute to the development of effective prevention and treatment strategies for COVID-19, MIS-C, and long COVID syndromes in children.

## Introduction

SARS-CoV-2 infection in children occasionally leads to severe multisystemic inflammatory syndromes (MIS), including MIS-C and severe pneumonia from COVID-19.

If most children who contract SARS-CoV-2 experience no or only mild symptoms, why do some develop severe hyperinflammation? Could MIS-C be considered a new disease? Many viruses can cause hyperinflammation, and viral myocarditis has been known for quite some time, so what makes MIS-C unique?

As pediatricians, we are always amazed by the level of active inflammation that can be present in patients with MIS-C and yet how quickly they respond to treatment. Inflammation can be tamed immediately compared to its activity, which is reminiscent of its epidemiology. It came out of nowhere, hit hard and now seems to disappear.

Post-acute sequelae of SARS-CoV-2 infection (PASC), more commonly known as long COVID syndrome, refer to a wide range of lingering symptoms that can occur weeks or months after an individual recovers from the initial acute phase of COVID. These prolonged effects are not limited to those who experienced severe cases, but have been reported in children with mild and even asymptomatic infections. Although MIS-C is a cytokine storm characterized by hyperinflammation that can occur in children infected with SARS-CoV-2, MIS-C has also been associated with long COVID symptoms, and recent research has suggested that MIS-C may be a more severe extension of long COVID syndrome. Furthermore, MIS-C can be classified as PASC since these syndromes present 2 to 6 weeks after SARS-CoV-2 infection.

This paper will review the current literature, focusing on the immunological aspects of MIS-C and severe COVID pneumonia in children and its potential link to long COVID.

### Epidemiology of SARS-CoV-2 infection in children

Approximately 18% of all coronavirus infections occur in children, and around 1% of COVID-19 patients are children. The incidence of COVID-19 was higher in ages 5–15. Seropositivity in the United States of America is around 75% for children, which is higher than that described in adults. Fortunately, in most pediatric cases (> 90%), SARS-CoV-2 infection is asymptomatic or presents only mild symptoms, such as low fever, dry cough, and weakness. Sixty-six percent of all infected children show no symptoms, 27% have mild flu-like symptoms, 5% have moderate (but pneumonic) symptoms, and only 2% are severe enough to require ICU care (Forrest et al. 2022).

Approximately 48 of every 100,000 children under 18 years of age are hospitalized annually due to SARS-CoV-2. This number is highest for children between the ages of 0 and 4 years (66.8 per 100,000), followed by those aged 12 to 17 years (59.9 per 100,000), and for those aged 5 to 11 it is the lowest (25 per 100,000 cases). During the Omicron wave, hospitalization rates between 0 and 4 years were 15.6/100,000. Children aged 5–11 who did not receive the vaccine had a rate of 19/100,000, while those who received the vaccine had a much lower rate of 9/100,000. Although hospitalization rates for SARS-CoV-2 and influenza are similar for children under 12, those over 12 are more likely to be hospitalized due to SARS-CoV-2 infection (Marks et al. 2022; Shi et al. 2022). Patients who are considered high-risk have a higher likelihood of being hospitalized. However, it is not entirely clear if this is due to the severity of their illness or if hospitals are quicker to accept high-risk patients (Shane et al. 2020). We now understand that the hospitalization rate is overestimated because general testing does not differentiate between those hospitalized due to COVID and someone who incidentally tests positive (Beck and Gandhi 2021).

Deaths from COVID-19 in children have been rare, ranging from 0.17 per 100,000 people as of February 2021, in seven countries, including the United States, the UK, Italy, Germany, Spain, and France. In fact, of the total estimated mortality from all causes in an average year, only 0.48% were attributed to COVID-19 in children under 18 years of age (Bhopal et al. 2021).

Although the mortality rate for COVID-19 in children is low, it is also evident that the virus does not discriminate according to age. In addition, even young people who were healthy before may need supportive treatment. On a positive note, most experts believe that children usually experience milder symptoms when infected with the SARS-CoV-2 virus.

### Cytokine storm and beyond

**Fig. 1:**
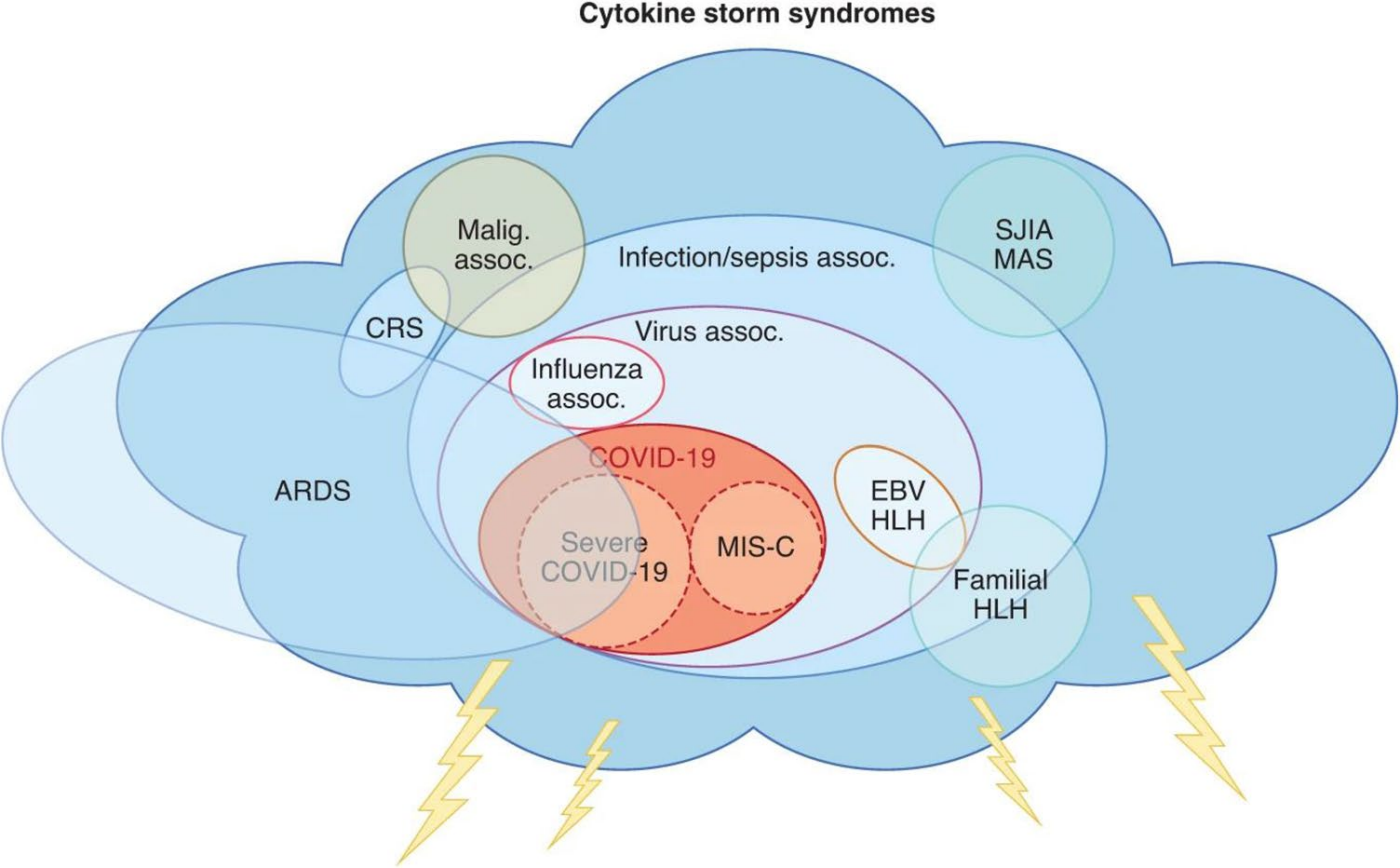
Cytokine storm syndromes (figure adapted from ref. (Buszko et al. 2021)). (*Assoc* association, *CRS* cytokine release syndrome, *EBV* Epstein–Barr virus, *HLH* hemophagocytic lymphohistiocytosis, *Malig*, malignancy, *MAS* macrophage activation syndrome, *SJIA* systemic juvenile idiopathic arthritis.)

There are several stages of COVID-19 that determine the treatment strategy. Stage 1 is an early viral infection, with symptoms of fever, respiratory or gastrointestinal, and lymphopenia. Phase 2a is a nonhypoxemic pulmonary phase, while phase 2b is hypoxemic. Finally, the third stage is multisystemic inflammation syndrome (MIS), which is sometimes accompanied by a cytokine storm as a characteristic of the pathogen (Fig. 1). A real cytokine storm occurs only in a small proportion of patients – 2% of all patients and 8% to 11% of severe cases. (Szekanecz et al. 2022). (At first, after looking at the China reports from early 2020, it seemed that hyperinflammation was the main reason for the high mortality rate. However, we are now sure that what we are facing is a localized cytokine ‘flooding’ instead of a systemic 'storm' (McGonagle et al. 2021).) This late stage of COVID-19 is also characterized by the activation of bradykinin storms, coagulation and complement cascades, endotheliitis, vascular leaks and edema, microthrombotic events, and extracellular neutrophil traps (Szekanecz et al. 2022).

Lymphopenia is often the first laboratory sign of SARS-CoV-2 infection. The number of circulating CD4 + and CD8 + T cells decreases significantly in COVID-19. The degree of lymphopenia is more pronounced in the elderly and those who require intensive care and correlates with mortality (Mathew et al. 2020; Wang et al. 2020). It is not clear, but the virus might directly affect these cells. As a result of increased chemotaxis, lymphocytes are redistributed toward tissues (e.g., lung interstitium) occurs. Interleukin-6 (IL-6) and other cytokines also inhibit physiological T cell activation (detected by the coexpression of CD38 and HLA-DR coexpression). Finally, T cell lymphopenia is also driven by apoptosis: surviving T cells in SARS-CoV infections have an "exhausted" dysfunctional phenotype showing increased expression of programmed death 1 (PD-1) expression (Diao et al. 2020). In addition to the maladaptive immune response of T cells, patients with severe COVID pneumonia showed a significant increase in the number of plasma cells (Mathew et al. 2020).

Not only does the adaptive immune system play a role in COVID-related hyperinflammatory syndromes, but autoinflammation has also been shown to contribute (Moga et al. 2022). Viral recognition within infected cells activates the NLRP3 inflammasome and NF-κB signaling, which is also a reason for the excessive production of pro-inflammatory cytokines and chemokines (Sefik et al. 2022). Pyroptosis is an inflammatory type of programmed cell death that occurs in response to infection or damage. After activating the NLRP3 inflammasome, which accompany the autoinflammatory response, caspase-1 cuts the N-terminal part of gasdermin D. This cleavage part is responsible for pyroptosis because these particles create a pyroptotic pore in the cell membrane and cytokines are released through them into the extracellular space. Furthermore, pyroptosis further enhances the production of IL-1-like cytokines through positive feedback. The role of pyroptosis in the release of numerous inflammatory mediators (IL-1b) has also been confirmed in COVID-19 (Yang 2020; Zheng et al. 2021).

### Why is COVID-19 rare and mild in children?

COVID-19 is generally mild in children. Hospitalization rates for COVID-19 are highest in the elderly, but why? One likely explanation is thymic involution. A research team has shown that the incidence of hospitalizations for COVID-19 in several countries consistently doubles every 16 years. They concluded that the risk of hospitalization for COVID-19 increases exponentially with age, inversely proportional to the production of T cell production (Palmer et al. 2021).

Furthermore, the hospitalization rate for COVID-19 is higher in men than in women. Men have a higher rate of thymic involution than women and also produce fewer T cells. Therefore, the hospitalization rate for COVID-19 may be due, at least in part, to thymic involution. However, the authors also found that other factors protect the under-20 age group from severe disease by an additional 49–75%. Therefore, thymic involution is one, but not the only reason, for the increase in the hospitalization rate with age (Palmer et al. 2021).

An area of active research is the role of cross-reactive immunity against other viruses, such as the common cold. (We can assume that exposure to other coronaviruses may provide some level of protection against COVID-19, but research proved otherwise: children after prior COVID-19 or MIS-C showed some loss of cross-neutralization against all variants, with the most pronounced loss against Omicron (Tang et al. 2022).) Hypothetically, the nonspecific immunomodulatory effect (or ‘‘off-target’’) effect of vaccinations containing live pathogens in childhood can also protect against future SARS-CoV-2 infections. Furthermore, there is evidence that viral interference in the mucosa may play a role (Piret and Boivin 2022). The mechanism involved in viral interference between prior and subsequent SARS-CoV-2 infection is mediated by interferon pathways (Fage et al. 2022).

The respiratory mucosa of young children is quite different from that of adults (Pierce et al. 2021). Young children's mucous membranes are penitent for type 1 interferon responses, which are essential to mediate local defenses against SARS-CoV-2 infection (Loske et al. 2021; Yoshida et al. 2021). Generally, the innate immune response is stronger in children, whereas the adaptive immune system is less developed and immature than in adults (Yoshida et al. 2021). When the virus is quickly cleared, it can help prevent the overwhelming immune response known as a cytokine storm. As a result, young children can better combat SARS-CoV-2 infection and avoid COVID-19. However, as we age, our mucosal surfaces become less effective in producing these responses (lower activation before infection, with weaker induction of interferon signaling after infection), making us more susceptible to SARS-CoV-2 infection (Loske et al. 2021).

Finally, other risk-reducing factors are more commonly known. For example, smoking is less prevalent during childhood, and children's airways aren’t exposed to pollution for as long as adults. Comorbidities that occur later in life, such as type 2 diabetes mellitus or high blood pressure, are not as prevalent in children. (The results of a systematic review and meta-analysis showed that certain independent risk factors in childhood increase the probability of severe COVID. These include newborn age, chronic lung disease, diabetes, premature birth, heart disease, and immunosuppressed state. However, asthma, neurodevelopmental disorders, and age less than 3 months are not associated with increased severity (Choi et al. 2022).)

#### Are the dangers of severe childhood COVID-19 still on the decline?

The emergence of the more contagious Omicron variant has presented us with numerous difficulties, notably its increased transmissibility, reduced effectiveness even after vaccination, and waning efficacy over time. Fortunately, the pandemic that had previously spared children from its full effects prevented it from becoming even worse. Studies have shown that the effectiveness of the Omicron vaccine decreases with time; however, the Omicron wave resulted in significantly milder acute cases. Furthermore, studies, including a very recent meta-analysis, indicate that complete vaccination is still essential to reduce emergency department visits and urgent care encounters associated with COVID-19 infection in children (Klein et al. 2022, Gao et al. 2023).

In conclusion, the mRNA vaccine is also effective against the Omicron variant in young people. Among partially vaccinated children who received an incomplete series of vaccinations, the vaccine was 13.6% effective against infection and 42.3% effective against hospitalization. In contrast, those who received a complete series of vaccinations had an efficacy of 36.8% for infection and 82.7% for hospitalization. Protection against hospitalization gradually decreases over time; after 20 weeks, protection is only 76% (Tan et al. 2022).

Immunity levels are high after infection, but, similarly to vaccinations, this immunity can fade over time. In cases where the person was not vaccinated and contracted the omicron virus, the said person was still protected from reinfection 90.7% of the time within a two-month period and 62.9% of the time after 4 months had passed. Research shows that vaccinated children who have had Omicron infection are protected against reinfection 94.3% of the time within 3 months and 79.4% of the time 4 months after vaccination. As it stands, this ‘‘hybrid’’ immunity offers the greatest protection. Of the 887,193 children included in a large cohort study that was already tracking the dominant spread of Omicron, 193,346 were infected. Of the infected, 103,338 had the Omicron strain. Of the 309 total hospital admissions, 294 were not vaccinated. The seven deaths occurred among unvaccinated children (Tan et al. 2022).

### MIS-C is a distinct disease

In April 2020, UK health officials reported the first cases of what would become known as multisystem inflammatory syndrome in children (MIS-C). The affected children had various symptoms that suddenly appeared and then progressed rapidly. As the word of the syndrome spread throughout the world, more and more cases began to be reported. Symptoms of MIS-C include fever, abdominal pain, vomiting, diarrhea, and rash. In severe cases, MIS-C can cause organ damage and even death. MIS-C has clinical features that overlap with Kawasaki disease, including fever, rash, and lymphadenopathy. However, MIS-C also often includes additional features, such as gastrointestinal symptoms, cardiac involvement, and neurological manifestations. The primary age group affected by Kawasaki disease is children under five years of age, while MIS-C predominantly affects children aged 2 to 14.

The cytokine profiles of adults with acute COVID-19 disease differ entirely from those seen in children with MIS-C or Kawasaki disease. IL-8 is a chemokine associated with lymphopenia in severe cases of COVID-19, and IL-7 is a cytokine involved in the maintenance of T cells and is associated with lymphocyte counts. IL-8 and IL-7 were elevated in adults with acute COVID-19. Compared to MIS-C and Kawasaki disease, both showed only slight elevations in these cytokines, indicating that the hyperinflammatory state in these conditions overlaps in part, but differs from that seen in acute COVID-19. However, a difference was also found in the immunopathology of Kawasaki disease and MIS-C. In MIS-C, the level of IL-17A, which we know plays an essential role in Kawasaki disease, was significantly lower (Consiglio et al. 2020). The main way to distinguish MIS-C from Kawasaki disease is by elevated concentrations of CXCL9. The stratification of patients with MIS-C based on their high or low CXCL9 concentrations indicates that those with severe MAS have a similar pathophysiology (Rodriguez-Smith et al. 2021). MIS-C is also unique in that it features an expansion of the expansion of polyclonal Vbeta21.3 T cells in both the CD4 and CD8 subsets not directed against antigenic peptides of SARS-CoV2, which was not detected in patients with KD, TSS and acute COVID-19 (see details later). Furthermore, the cytokine storm they detected was correlated with this (Moreews et al. 2021).

By analyzing signatures with the help of artificial intelligence, researchers have recently found that though both KD and MIS-C produce a similar immune response guided by IL-15 / IL15RA, they differ quite extensively in other laboratory parameters and cardiac phenotypes. Furthermore, the response is more intense in MIS-C cases. Their results imply shared pathways of their proximal immunopathogenesis (Ghosh et al. 2022).

### Immunopathology of MIS-C

Based on complex immune profile studies, a characteristic pro-inflammatory cytokine profile is observed in MIS-C, which is associated with a significant increase in biomarkers related to type II IFN signaling (IFN-γ, CXCL9, and CXCL10), macrophage activation (IL-6, sTNFRI, IL-10, sCD25, IL-17, TNF-α, sCD163, CCL2, CCL3, CCL4, ferritin, and IL-15), chemotaxis (CXCL1, CCL3, CCL4, CDCP1) neutrophil activation (MPO and lactoferrin), endothelial injury and activation (VEGF, sVCAM-1/sCD106 and sE-Selectin/sCD62E) and mucosal immune dysregulation (CCL20, CCL28) (Gruber et al. 2020; Moreews et al. 2021; Sacco et al. 2022).

Immunophenotyping of peripheral blood cells in patients with MIS-C showed characteristic changes in the proportions and functions of immune cells. Naive CD4 + and CD8 + cytotoxic T cell counts were significantly reduced, as were the ratios of monocyte and natural killer (NK) cell ratios (Mathew et al. 2020). Furthermore, there was evidence of neutrophil and CD16 + monocyte activation (FcγR1) and increased expression of migration proteins (ICAM-1), all indicating increased flow of NK and myeloid cells to the periphery (Consiglio et al. 2020; Gruber et al. 2020). This is important because activated inflammatory cells that flow into organs establish the state of multiorgan inflammation in MIS-C. The abnormally low number of NK cells and CD8 + cytotoxic lymphocytes in circulation, combined with increased cytotoxic activation of these cells (as indicated by increased expression of the perforin and granzyme gene), contributes to maintaining inflammation, which also promotes the development of autoreactivity (Beckmann et al. 2021; Ramaswamy et al. 2021).

Autoantibodies have long been thought to play a role in Kawasaki disease, but the nature of their reactivity remains somewhat elusive. Recent studies profiling the autoantigen reactivity of MIS-C plasma revealed broadly reactive antibodies and novel candidates that recognize endothelial, gastrointestinal, and immune cell antigens. This finding suggests that while there may not be a single target for these antibodies, loss of tolerance of B cells is probably a contributing factor to the development of MIS-C (Gruber et al. 2020; Porritt et al. 2021a). Furthermore, increased plasmablasts and endothelium-reactive IgG are known features of severe MIS-C (Ramaswamy et al. 2021). Based on all of these studies, it can be concluded that a robust autoimmune signature can be observed in MIS-C.

**Fig. 2:**
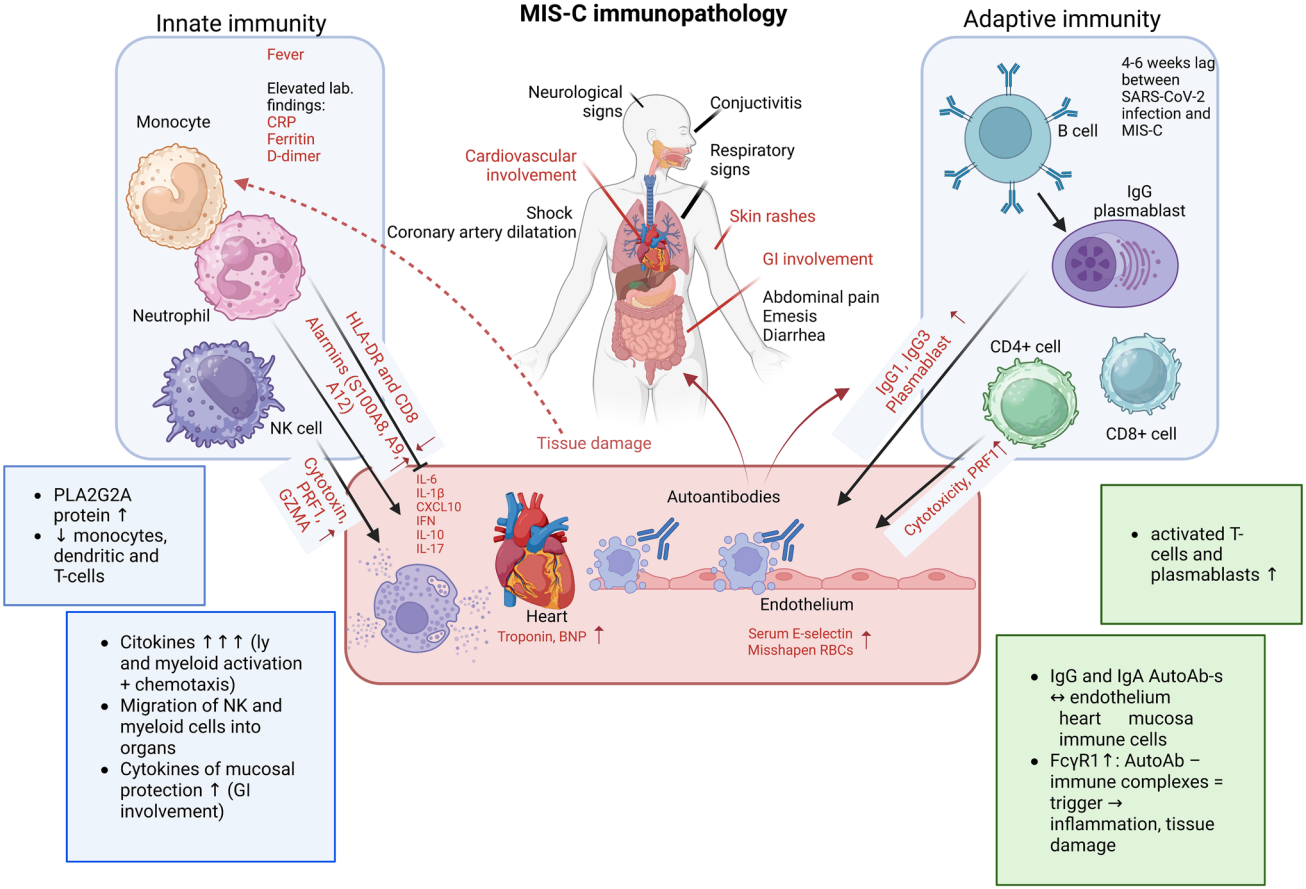
Immunopathology of MIS-C (figure adapted from ref. (Buszko et al. 2021)). *Ab* autoantibodies, *GI* gastrointestinal)

The immunopathology of MIS-C is summarized in Fig. 2.

#### The superantigen hypothesis

The concept of superantigens is the most common theory that explains the high cytokine release (storm) and tolerance of B cells. Various well-documented arguments favor it, such as the existence of a superantigen spike protein character and a skewed repertoire of T-cell receptors.

Based on computer modeling based on structure, we know that the SARS-CoV-2 spike protein contains a superantigen-like pattern. This pattern binds highly to the CD section of the variable domains of the alpha and beta chains of the T cell receptor (TCR) (Cheng et al. 2020). The superantigen motif is sequence and 3D similar to the Staphylococcus enterotoxin B superantigen, which also binds to TCR and CD28 and thus triggers toxic shock syndrome (TSS). Binding of the corresponding region of the SARS-CoV-2 spike protein is also followed by large-scale nonspecific T cell activation and proliferation, with massive production of the characteristic pro-inflammatory cytokine profile of TSS (Rivas et al. 2021). Furthermore, the superantigen-like region of SARS-CoV-2 can also activate B cells, stimulating them to produce autoantibodies.

Finally, analysis of the repertoire of T cells from patients with COVID-19 showed that severe COVID-19 disease is associated with a TCR-Vβ shift, the selection of specific Vβ genes, and increased J diversity, as observed during superantigen activation (Moreews et al. 2021; Porritt et al. 2021b). Interestingly, the TCR repertoires of patients with mild MIS-C are richer and more diverse than those of patients with severe MIS-C. The expansion of T cells with identical TCR-Vβ genes was correlated with the severity of the disease and the cytokine storm, indicating that it is an important mechanism underlying severe MIS-C (Porritt et al. 2021b). Furthermore, the repertoire of T cells returned to baseline within weeks after the resolution of the MIS-C resolution (Moreews et al. 2021).

#### Why is MIS-C delayed?

Usually, MIS-C occurs about a month after someone has contracted SARS-CoV-2. At that time, SARS-CoV-2 was already undetectable by the reverse transcription-polymerase chain reaction of the nasopharyngeal swab in most cases (Feldstein et al. 2020; Santos et al. 2021). If we consider that superantigens cause MIS-C, it will be reasonable that most antigens should be present during the early stages of infection (Han et al. 2021). So why does not MIS-C develop sooner? The most convincing explanation is that the virus can persist for a long time in children, particularly in the intestinal tract. The virus can be detected in children's feces long after infection, even after their nasopharyngeal tests became negative (Xu et al. 2020). The average time to viral clearance is nine days for the respiratory tract and 34 days for stool samples (Du et al. 2020). Furthermore, from biopsy samples, we know that the virus can be present in intestinal tissues for even longer (Arostegui et al. 2021). From an elegant study, we now also know that the gastrointestinal tract is not only a passive reservoir of virus, but also a source of viremia (consequential antigenemia), which can contribute to the development of MIS-C (Yonker et al. 2021).

#### Why is MIS-C rare?

The reason why only a minority of children infected with SARS-CoV-2 end up developing MIS-C is still unknown. It is plausible that the initial antibody response does not create enough neutralizing antibodies for some children. Consequently, the immune system overreacts if exposed again to the virus (Rivas et al. 2021). Another explanation (see more details later) for the susceptibility to MIS-C is genetics; some types of HLA may have a stronger reaction to specific viral antigens (Cheng et al. 2020). The latter is supported by the fact that the incidence of MIS-C varies by race and ethnicity (e.g. 50% of MIS-C cases in London were of Afro-Caribbean origin) (Whittaker et al. 2020; Stierman et al. 2021).

The role of immunity against previously experienced common cold coronaviruses (CCCs) is also discussed. In one study, anti-CCC antibodies were undetectable in patients with MIS-C, suggesting that infection with the SARS-CoV2 virus can trigger a stronger immune response without this type of protection (Consiglio et al. 2020). A recent study found that memory T cells generated in people previously infected with the CCC virus can eliminate SARS-CoV-2, which shares surface proteins with CCC. This suggests that these individuals have a more rapid immune response during the initial phase of viremia (Swadling et al. 2021).

It is worth mentioning that, according to the second hit theory, subsequent infections may be necessary to develop symptoms. According to second-hit theory, a pathogen (for example, during gastroenteritis) "hits" the window period after SARS-CoV-2 infection (Buszko et al. 2021). In another study, B cell reactions were compared; however, those with MIS-C showed a more developed antibody response upon presentation, which implies that they had experienced a postinfectious condition different from COVID-19 (Akindele et al. 2022).

#### How effective are SARS-CoV-2 vaccines against MIS-C?

Studies carried out in various countries have found that the prevalence of MIS-C is significantly decreasing but remains a serious complication of SARS-CoV-2 infection (Cohen et al. 2022; Levy et al. 2022; Miller et al. 2022; Sorg et al. 2022b; Whittaker et al. 2022). During the Delta and Omicron, the rate decreased by 73% and 86% in Germany, respectively, compared to the Alpha period (Sorg et al. 2022a). In southeast England, the rates of MIS-C per confirmed SARS-CoV-2 infection in children aged 0–16 years were 56% lower during the prevaccination delta (the period before a vaccine was available), 66% lower during the postvaccination delta (the period after a vaccine became available), and 95% lower during the Omicron period (Cohen et al. 2022). In a study in England, the estimated odds that MIS-C occurs after SARS-CoV-2 infection in those under 15 years of age were 0.045%, with a 95% credible interval ranging from 0.035 to 0.068% during the Alfa and Delta waves (Shingleton et al. 2022).

Several studies have found that not only has the prevalence decreased, but also the severity changed. Compared to the third, fewer reports of organ system complications, such as cardiovascular and respiratory problems, were published during the fourth wave of MIS-C. Hospital stays were shorter and the number of patients receiving ICU care was significantly lower in the fourth wave (Levy et al. 2022; Miller et al. 2022; Sorg et al. 2022a; Wang et al. 2022; Kenney et al. 2023). The severity of MIS-C severity can differ for a few reasons, such as host immune response variances, earlier clinical diagnosis, or MIS-C treatment. Furthermore, the clinical phenotype could be altered by having some degree of preexisting immunity conferred by SARS-CoV infection or COVID vaccination (Miller et al. 2022). Although there is no scientific evidence to support this hypothesis, the decrease in the risk of MIS-C may be attributed to significant mutations in superantigen motifs, which are believed to induce an exaggerated inflammatory reaction.

In the USA, a recent case–control study showed that the Pfizer BioNTech vaccine is 91% effective in preventing MIS-C among vaccinated individuals aged 12–18 years. Among the 102 patients with MIS-C cases, all 38 children requiring life support were not vaccined. Individuals aged 12–18 years who received two doses of the Pfizer-BioNTech vaccine were found to have a high level of protection against MIS-C (Zambrano et al. 2022).

Recently, there have been several reports of a hyperinflammatory condition (multisystem inflammatory syndrome after vaccination, MIS-V), similar to MIS-C, in children who have received the SARS-CoV-2 RNA vaccine. However, it is essential to put these reports in perspective. First, the incidence of MIS after receiving the SARS-CoV-2 vaccine is very low and is much less frequent in vaccinated children than in unvaccinated children. In the US, the reported rate for MIS after vaccination is 1 case per million individuals aged 12 to 20 years who received one or more doses (Yousaf et al. 2022). In France, the reported rate was 2.9 per 1 million 12–17 year-old vaccinated children (Ouldali et al. 2022).

Second, the symptoms of MIS-V are usually milder than those of virus-induced MIS-C, although many children still needed treatment in the intensive care unit. The median age of those affected was slightly older (the median age was 16 years in the US), and a specific phenotype was described: Patients were diagnosed more frequently with cytolytic hepatitis (20–50%) and some showed transient hypereosinophilia (20%). Laboratory symptoms of inflammation were less alarming than expected, such as a lower CRP level and a normal hemoglobin level. All cases have recovered, and no death is known (Ouldali et al. 2022; Yousaf et al. 2022).

Finally, there is no direct evidence that the vaccine would cause MIS; instead, it appears to be a coincidence that some children who develop MIS-C have also been vaccinated. Some patients contracted SARS-CoV-2 and later developed MIS-C while simultaneously receiving vaccines, which could easily have been misinterpreted as MIS-C associated with vaccination. Furthermore, most of the vaccinated children who later developed MIS-C showed clear evidence of breakthrough infection with SARS-CoV-2 (Cole et al. 2022; Yousaf et al. 2022). Only 0.3 cases of MIS per million vaccinated individuals have been reported in children without evidence of previous SARS-CoV-2 infection (Yousaf et al. 2022). In France, 8 out of 12 cases did not show evidence of the last infection (Ouldali et al. 2022).

In conclusion, although MIS-C is a severe disease that should be monitored, it is clear that the risks posed by the SARS-CoV-2 RNA vaccine are far outweighed by its benefits.

### Should children receive the SARS-CoV-2 vaccination after MIS-C?

Although mRNA vaccines are effective in young people, it is still unclear whether SARS-CoV-2 vaccination is safe in patients who previously experienced MIS-C. As mentioned above, MIS-C shares similarities with illnesses caused by exposure to superantigens, much like toxic shock syndrome. A key question arises in light of MIS-C cases among children: Could reexposure to viral antigens cause relapse? This fear is only compounded by recent reports of rare but severe inflammatory events after SARS-CoV-2 vaccination, such as myocarditis in young men and more general multisystem inflammation (MIS-V).

To date, only two international surveys and a short-term, non-controlled study have been conducted to assess the safety and immunogenicity of SARS-CoV-2 vaccines in children with a history of MIS-C. Fortunately, all found reassuring answers.

To assess the acceptance of SARS-CoV-2 vaccination in children who have previously suffered from MIS-C, Levi Hoste and his research team surveyed a global population of pediatric rheumatologists and infectious disease specialists. After analyzing the survey results, it was discovered that most centers did not vaccinate those with a history of MIS-C due to limited safety and efficacy information. However, 17% of suitable patients were vaccinated and only experienced mild or moderate adverse effects without significant long-term problems (Hoste et al. 2022).

In a survey conducted internationally, Minoia et al. analyzed the perspectives of pediatric rheumatologists on the management of children with previous MIS-C cases with respect to COVID-19 vaccinations and other immunizations. The survey was distributed to 288 pediatric rheumatologists and 111 of them responded with a response rate of 38.5%. Compared to Hoste et al., the survey results were more encouraging: Most of the respondents recommended vaccination with COVID-19 for children with a history of MIS-C (84%). Although a waiting period of three months after the acute stage of MIS-C is generally preferred, there has not yet been a definitive consensus on when exactly the SARS-CoV-2 vaccine should be administered. Sixty-seven of the total of 228 centers (28%) in 22 countries had already vaccinated patients with MIS-C with MIS-C, where 89% did not report any adverse reactions. Unfortunately, six cases reported complications such as unspecified diseases in two children, mild symptoms in three children, and one case even registered a reaction similar to that of MIS-V. The overwhelming majority of centers wanted to maintain their current vaccination practices with non-live (99%) and live (93%) vaccines; however, the desired timelines varied tremendously (Minoia et al. 2022).

Howe et al. published a brief study on the safety and immunogenicity of the Pfizer-BioNTech COVID-19 vaccine in children with a history of MIS-C. The study enrolled 23 children aged 12–15 who had recovered from MIS-C and received the two-dose regimen. The study found that the vaccine was well tolerated and no significant adverse events were reported. They found that all participants developed detectable antibody responses after the second dose. Although the study has limitations, such as the small sample size and the lack of a control group, it suggests that the Pfizer-BioNTech COVID-19 vaccine is safe and immunogenic in children who have recovered from MIS-C (Howe et al. 2022).

### Immunopathology of COVID-19

A study published in Science showed that a subset of patients with COVID-19 produce autoantibodies that can block type I interferons (alfa and omega) (Bastard et al. 2020). The authors investigated the presence of these autoantibodies in a group of patients hospitalized in the ICU and in a control group of healthy individuals. They found that more than 10% of the ICU patients had high titers of these antibodies (20% over 80 years), while none or low levels were observed in patients with asymptomatic or mild infection (Bastard et al. 2020, 2021). The presence of these autoantibodies means a higher risk of serious disease than other classical risk factors, except age (Bastard et al. 2021). They also showed that when the effect of type 1 interferons is blocked, there is more viral replication in vitro. Furthermore, they found that patients with autoantibodies against IFNα2 have low or undetectable levels of circulating IFNα concentrations in vivo. Others found that these autoantibodies also affect type I IFN activity in peripheral blood mononuclear cells (Wijst et al. 2021).

The most exciting part is that the researchers discovered that these autoantibodies had already existed before infection, although only a few patients were tested (Bastard et al. 2020). In conclusion, the authors suggested that the production of autoantibodies against type I interferons contributes to the severity of COVID-19. In other words, this means that infection did not induce the appearance of autoantibodies; rather, their presence led to severe COVID-19 disease.

Interestingly, more than half of the patients were elderly (> 65 years old), and almost all were men (95%) (Bastard et al. 2020). In the healthy and uninfected population, the frequency of occurrence of these autoantibodies is rare under the age of 65, but increases sharply above the age of 65 (Bastard et al. 2021). These findings help explain why more men are likely to be severely affected by COVID-19 and how the risk of severe disease increases with age (Bastard et al. 2020). Subsequently, several research groups around the world verified their initial observations.

Many patients with COVID-19 pneumonia with low oxygen levels show a range of different autoantibodies. These were probably caused by the SARS-CoV-2 infection and may have affected the progression of the disease (Wang et al. 2021). This, along with a study of a small group of patients over time, suggests that SARS-CoV-2 infection increases the levels of type I IFN autoantibodies that already exist (Shaw et al. 2021). These autoantibodies were found in children and were also an independent risk factor for developing severe (hypoxemic) COVID-19 pneumonia (Bastard, personal communication, 2022).

According to research by Bastard et al. the presence of these antibodies can even suppress vaccine effectiveness. They discovered that even after two doses of the mRNA vaccine and the existence of circulating antibodies that could neutralize SARS-CoV-2, autoantibodies that block type I IFN may be behind a large percentage of hypoxemic COVID-19 pneumonia incidents (Bastard et al. 2022).

### Genetics of COVID-19 and MIS-C in children

Although we know several health conditions that make people more vulnerable to severe COVID-19, it is still a mystery why some otherwise healthy individuals, including young adults and children, develop deadly complications from SARS-CoV-2 infection. In both children and adults, a comprehensive evaluation of candidate genes involved in innate immunity (including those that encode pattern recognition receptors and their downstream mediators), antigen presentation, cytotoxicity, costimulation, inflammation, and thrombosis/hemostasis is warranted, given the role each plays in the pathogenesis of COVID-19. Because MIS-C and CSS share some characteristics, the same genes that contribute to HLH may also play a role in the development of MIS-C.

#### Primary immunodeficiencies (PIDs)

Interestingly, patients with antibody deficiencies are not susceptible to severe COVID pneumonia, and the general course of SARS-CoV-2 infection appears similar to that of the general population. This agrees with what has been found for critical influenza pneumonia, which explicitly affects patients with inborn errors of type I IFN immunity but not others, even those lacking T and B cells (Zhang et al. 2022a).

Much less is known about the relationship between PIDs and MIS-C. Several small studies have proposed possible monogenic reasons for MIS-C. Genes related to immune dysregulation, such as SOCS1, XIAP, CYBB and TBK-1, have been found in patients with MIS-C (Schulert et al. 2021).

Malle et al.' study reveals that SARS-CoV-2 can trigger an atypical inflammatory syndrome in infants with Down syndrome (DS). This not only emphasizes the idea that children with DS are vulnerable to atypical disease presentation, but the authors also showed that uninfected children with DS have a significantly altered (and ‘‘alerted,’’ pro-inflammatory) immune state. Their findings suggest that children with DS are prone to MIS-C due to inherent genetic factors rather than just exposure to pathogens (Malle et al. 2021).

#### HLA Association

The expansion of TRBV11-2 has been associated with HLA class I alleles (Porritt et al. 2021b). This was recently supported by evidence that the combination of HLA-A*02, B*35 and C*04 alleles increased the risk of developing MIS-C (Sacco et al. 2022).

#### Interferon deficiency and severe COVID-19 pneumonia

The production of type I IFNs by respiratory epithelial cells and plasmacytoid dendritic cells is necessary for host defense against SARS-CoV-2. However, inadequate type I IFN immunity in the respiratory tract, which can depend on age and sex, can lead to viral spread and eventually to disease (pulmonary or systemic inflammation). Interferon deficiency can be due to either a lack of production or an inability to respond to the protein.

Most patients with type 1 autoimmune polyendocrine syndrome (APS-1), caused by biallelic mutations in the AIRE gene, have autoantibodies against IFN-α and IFN-ω. Many of these patients had contracted severe COVID-19 and some even died. In particular, several individuals were asymptomatic despite being infected with SARS-CoV-2 (Beccuti et al. 2020; Bastard et al. 2021; Meisel et al. 2021).

A research group established the COVID Human Genetic Effort (www.covidhge.com) to test whether life-threatening COVID-19 in some patients may be caused by genetic disorders that affect immunity to SARS-CoV-2. They analyzed the exomes or genomes of 659 patients with severe COVID-19 pneumonia and 534 with asymptomatic or mild infections. At least 3.5% of patients with life-threatening COVID-19 pneumonia had genetic defects in TLR3- and interferon regulatory factor 7 (IRF7)—dependent induction and amplification of type I IFN. These findings suggest mutations in other type I IFN–related genes in patients with severe COVID-19 (Beccuti et al. 2020). Since this publication in 2020, it has been a fact. The deficiencies of autosomal Toll-like receptor 3 (TLR3) and TLR7 linked to the X chromosome are inborn errors of type I interferons that are found in around 1% to 5% of younger patients (under 60 years old) with critical pneumonia, while a lower proportion of older patients have them (Zhang et al. 2022a). Deficiency of the Type I interferon receptor and TANK binding kinase (TBK-1) can lead to serious and potentially life-threatening diseases (Zhang et al. 2020; Khanmohammadi et al. 2021; Schmidt et al. 2021). A recent study found that patients with TLR7 deficiency accounted for approximately 1.8% of male patients with severe COVID-19 pneumonia below the age of 60 years. Because this gene is located on the X chromosome, it helps explain why younger men are at higher risk of developing serious and critical forms of the disease than women (Asano et al. 2021). After conducting an exome-wide gene burden analysis to find the rare variants responsible for critical COVID-19, no single gene had statistical significance for GW. However, when adopting a recessive inheritance model, X-linked TLR7 was identified as having the strongest association (Matuozzo et al. 2022).

Inborn errors of immunity were more common in children than in adults, with more than 10% of hospitalized children with severe COVID-19 displaying genetic deficiencies in several genes related to type I interferon pathways (Zhang et al. 2022b). For context, these same deficiencies were not found among 1000 + mildly infected young people with SARS-CoV-2 who did not develop pneumonia. In other words, around 10% of hospitalizations for COVID-19 pneumonia are estimated to be due to IFN type I immunity deficiencies. (As children usually have a more robust innate immune response than adults—and fewer neutralizing autoantibodies -it was expected that a higher proportion of hospitalized children have a predisposing genetic factor.)

#### Genetics of Kawasaki disease

Kawasaki disease (KD) is tentatively linked to genetic and environmental influences. A familial tendency toward the disorder and its higher prevalence in Northeast Asia indicate a possible genetic disposition leading to KD. In fact, some studies have shown that specific genetic variants can make individuals more susceptible to the disease (Onouchi et al. 2012, 2016). However, there is no single mendelian cause of Kawasaki disease. Instead, several genes are likely to contribute to its development. A systematic review article found that the following gene polymorphisms may play a role in susceptibility to KD: ACE, BLK, CASP3, CD40, FCGR2A, FGβ, HLA-E, IL1A, IL-6, ITPKC, LTA, MPO, PD-1, SMAD3, CCL17, and TNF (Xie et al. 2018). Four main groups were classified when identifying susceptibility genes using GWAS and the candidate gene approach: those associated with enhanced T cell activation (ITPKC, ORAI1, STIM1), dysregulated B cell signaling (CD40, BLK, FCGR2A), decreased apoptosis (CASP3), and altered transforming growth factor β signaling (TGFB2, TGFBR2, MMP SMAD) (Kumrah et al. 2020). Furthermore, others revealed that KD is associated with HLA class 1 antigen genes (Kumrah et al. 2020; Lo 2020; Chen et al. 2021). By analyzing adult people’s DNA, whole exome sequencing revealed 12 uncommon and potentially pathogenic variants within genes related to autophagy, Kawasaki disease, restriction factors and immune responses (Ronit et al. 2021).

#### Cytokine storm genes in COVID-19 and MIS-C

As demonstrated above, COVID-19 pneumonia and MIS-C can lead to cytokine storm syndrome. Heterozygous mutations related to familial (primary) HLH can often also be found in other CSS with different underlying causes (Bami et al. 2020; Eloseily et al. 2020). An increasing number of studies have shown that heterozygous variants in HLH-related genes may be related to SARS-CoV-2-related CSS (Table 1). Recent studies using computational algorithms and multilayered transcriptomics have concluded that the pHLH genes could interact with SARS-CoV-2, predisposing patients to severe COVID-19 through a neutrophil activation signature similar to that described earlier in HLH (Ding et al. 2021; Schimke et al. 2022). Another study suggests that the UNC13D and AP3B1 variants (known as HLH genes) might be associated with the development of severe cytokine storms, critical disease, and fatal outcomes in patients with COVID-19 (Luo et al. 2021).

**Table 1:** Immune gene mutations associated with MIS-C (table adapted from ref. (Schulert et al. 2021; Cron 2022; Sacco et al. 2022; Vagrecha et al. 2022)

Gene mutations	Immune pathway affected	Immune disorder
Genes related to innate immunity		
SOCS1	Cytokine signaling FN regulation	Immune dysregulation PID Inflammasomopathy Interferon b1 deficiency defect in IFNa and IFNb induction
XIAP	Apoptosis	
CYBB	Microbicidal oxidation	
TBK-1	Antiviral responses IFN production	
NLRC4	Inflammasome	
IFNB1	Interferon b1	
TLR3	Toll-like receptor 3	
MHC class I genes		
HLA-A*02, HLA-B*35 HLA-C*04	Viral recognition T-cell activation	Dysregulation of T-cell responses NK cell activation
fHLH genes		
PRF1	Perforin	Deficiency in perforin-mediated cytolysis
LYST	Vesicle sorting	
AP3B1	Vesicle transport	
DOCK8	Vesicle transport	
UNC13D	Vesicle priming	
STXBP2	Vesicle fusion	
STX11	Vesicle fusion	

Anshul Vagrecha and Randy Cron (2022) led a research group that reported rare missense heterozygous variants of the HLH genes in their cohort of children with MIS-C. They identified 54 rare variants in immune regulatory genes from patients with MIS-C. Twenty-nine patients (74.3%) had at least one genetic variant. The most common variants were missense mutations in the DOCK8 gene that were found in four patients (10.2%). Furthermore, of the 39 patients, six (15.3%) had rare heterozygous missense mutations in pHLH-related genes (LYST in two patients; STXBP2, UNC13D, PRF1, and AP3B1 in each). Furthermore, a patient had a missense variant located in NLRC4, an autoinflammatory gene linked to CSS in infants with dominant missense mutations. Interestingly, although none of the children in the control group had any genetic variants in the pHLH genes or DOCK8, a quarter of those with MIS-C tested positive for at least one variant (Vagrecha et al. 2022).

Here, we must mention that the frequency of occurrence of risk alleles varies significantly from one geographical region to another. A recent publication by Japanese researchers stated that those under 65 years of age who had the DOCK2 genetic variant were more likely to experience severe symptoms of COVID-19. This allele is common among East Asians, but rare compared to Europeans (Namkoong et al. 2022).

#### The probability of MIS-C occurring in siblings is very low

The probability of KD is higher in close relatives of patients who have already been diagnosed with the disease. In fact, siblings of patients with KD are ten times more likely to develop KD themselves (Chen et al. 2021).

In sporadic cases, there is a family accumulation of MIS-C. For example, two siblings in one family became ill and one member of a monozygotic twin pair was affected. The fact that the other member of the twin pair contracted SARS-CoV-2 but remained healthy is interesting (Lim et al. 2021).

#### Certain ethnic groups are disproportionately affected by MIS-C

It is a known fact that there are racial and ethnic disparities in the incidence of Kawasaki disease. Research suggests that non-Hispanic Asian populations experience the highest rate, with non-Hispanic Black populations following closely behind. In comparison, MIS-C was more prominent among Hispanic and non-Hispanic Black children in the US, whereas it occurred less often among non-Hispanic White and non-Hispanic Asian children. Even after accounting for the existing disparities in COVID-19 infections and geographic differences, these variations in MIS-C cases between different ethnicities remain (Stierman et al. 2021). This reveals that the cause of this phenomenon goes beyond mere infection rates. Social determinants of health could be a key factor in this, but they are clearly not the only one. That is, another study found that the risk that black and Latino children develop MIS-C is much higher than the overrepresentation of black and Latino adults for both severe COVID-19 and COVID-19-related mortality. In the case of adults, this difference is generally explained by preexisting health disparities. However, if health disparities were solely responsible for overrepresentation among children, we would expect them to be at a similar level. Evidently, other crucial factors are also more likely to be involved, such as genetic predisposition to a hyperinflammatory response (Middelburg et al. 2021).

### Conclusion, immunopharmacological implications

The COVID-19 pandemic has caused a global public health crisis, with almost a billion confirmed cases and millions of deaths. The disease spread rapidly, putting pressure on healthcare systems. Rigorous scientific studies are being conducted to explore a multitude of preventive and curative treatments, such as vaccines, antibodies, or immunosuppressants (Hasan et al. 2021).

Research suggests that hyperinflammatory syndrome affects the severity of COVID-19. Currently, evidence indicates that hyperinflammatory syndrome is caused by a dysregulated innate immune response of the host. According to the two-step general model of the pathogenesis of life-threatening COVID-19, insufficient type I IFN immunity during the first days of infection leads to viral growth and spread. This causes pulmonary and systemic inflammation that can damage (Zhang et al. 2020).

Although more research is needed to understand the full extent of this phenomenon, the implications could be far-reaching for our understanding of disease pathology and treatment in the future.

#### Antibodies

Type I IFN autoantibodies are estimated to have caused nearly 1 million deaths from COVID-19 worldwide. With this in mind, future research on these antibodies may have significant clinical practicality. First, people (especially people with risk factors for severe disease) can be tested for autoantibodies with a simple blood test before any potential SARS-CoV-2 infection. Those who return positive should be vaccinated as soon as possible and given priority for booster shots. It is also possible to specifically test during the early stages of COVID-19 because more targeted treatments—like IFNβ, neutralizing monoclonal antibodies against SARS-CoV-2, or plasma exchange—could be beneficial in patients with autoantibody positive patients (Zhang et al. 2022a). Clinically relevant approaches are being developed to quickly recognize people with positive interferon antibody responses so that subsequent clinical interventions can be administered (Akbil et al. 2021).

#### Genetics

By understanding which genes can contribute to pediatric COVID-19 and MIS-C, clinicians can make more informed treatment decisions. Children with mutations associated with HLH may benefit the most from therapies targeting excessive inflammation, such as glucocorticoids, cytokine blockers (e.g., IL-1 and IL-6), and lymphocyte-targeted treatments (e.g., calcineurin inhibitors). In comparison, children infected with SARS-CoV-2 who have genetic disorders in their innate immune responses, including type I IFN, may improve with treatment that includes recombinant interferon. This will help control viral replication and prevent cytokine storm syndrome from occurring (Schulert et al. 2021).

#### MIS-C

Fortunately, treatments for MIS-C are available and have been shown to be effective. The prognosis for patients with MIS-C is excellent and only a few patients have experienced long-term sequelae. Furthermore, both national and international registries for MIS-C cases have recently found a lower occurrence rate, possibly due to the different effects of the SARS-CoV-2 variants or vaccinations.

The recurrence of KD recurrence is uncommon and we are optimistic that the same will be true for MIS-C. However, autoantibodies in patients with MIS-C suggest a potential for relapse or other disorders related to autoimmune characteristics. Although inflammation is short-lived, these autoantibodies could still pose a risk.

In conclusion, although MIS-C can be managed with available treatments, more scientific research is still required to fully understand the condition (Gruber et al. 2020).

#### Implications for Long COVID

Long COVID refers to the persistence of symptoms related to infection with the SARS-CoV-2 virus (Soriano et al. 2022). Specific symptoms and their duration can vary widely between individuals, but common symptoms of long COVID include fatigue, shortness of breath, chest pain, coughing, joint pain, headache, difficulty sleeping, brain fog, and loss of taste or smell sense. Other less common symptoms can also occur, such as gastrointestinal symptoms, heart palpitations, and skin rashes.

Long COVID can affect people of all ages, including children (Berg et al. 2022). Among children, the most frequently reported symptoms were fatigue, headache, arthromyalgia, chest tightness or pain, and dyspnea (Borch et al. 2022a; Pellegrino et al. 2022).

Research has shown that these long-term COVID symptoms cannot be attributed to any psychological effects of recent social restrictions in place (Borch et al. 2022b). Fortunately, long COVID symptoms are often temporary and improve in 1–5 months; however, they can still greatly reduce someone's quality of life.

Scientific evidence on the immunopathology behind the long COVID syndrome in children is still limited. However, some studies suggest that long COVID in children may be associated with an immune-mediated inflammatory response similar to what has been observed in adults with long COVID (Son et al. 2022). One hypothesis is that long COVID in children may be triggered by an autoimmune response. Unlike short-lived systemic problems caused by cytokine storms, autoantibodies are believed to trigger more specific and long-term damage and therefore may play a role in the pathogenesis of long COVID (Wang et al. 2021; Su et al. 2022; Zhang et al. 2023). The SARS-CoV-2 superantigen motif, which we have previously discussed in detail, could potentially contribute to the development of COVID-19 hyperinflammatory syndromes, such as MIS-C, and autoimmune reactions related to long COVID (Rivas et al. 2022).

In addition, there have been reports of neurological symptoms, such as brain fog and memory problems, that may be related to an autoimmune response. Women who have recovered from toxic shock syndrome regularly report persistent neurological symptoms such as headaches, impaired cognition, and memory loss (Rosene et al. 1982). These signs are strikingly similar to the neuropsychiatric problems described by those with long-term COVID. This provides further evidence that superantigens may be involved in the induction of long COVID. Furthermore, the recognition of neurotoxin patterns in SARS-CoV-2 opens up the possibility that inflammation caused by its spike protein could directly contribute to neurological symptoms associated with MIS-C and prolonged COVID (Rivas et al. 2022).In summary, gaining a clear and deeper understanding of the early and late immune response to SARS-CoV-2 infection could be a key factor in understanding the prolonged effects of long COVID.

## Data Availability

Enquiries about data availability should be directed to the authors
